# Experiences of gestational diabetes and gestational diabetes care: a focus group and interview study

**DOI:** 10.1186/s12884-018-1657-9

**Published:** 2018-01-11

**Authors:** Judith Parsons, Katherine Sparrow, Khalida Ismail, Katharine Hunt, Helen Rogers, Angus Forbes

**Affiliations:** 1 Adult Nursing, Florence Nightingale Faculty of Nursing, Midwifery & Palliative Care, King’s College London, James Clerk Maxwell Building, 57 Waterloo Road, London, SE1 8WA UK; 2 Department of Psychological Medicine, Institute of Psychiatry, King’s College London, London, UK; 3 Department of Psychological Medicine, Institute of Psychiatry, King’s College London, London, UK; 4 Diabetes Research Group, Diabetes & Nutritional Sciences Division, King’s College London, London, UK; 5 Diabetes Research Group, Diabetes & Nutritional Sciences Division, King’s College London, London, UK; 6 Adult Nursing, Florence Nightingale Faculty of Nursing, Midwifery & Palliative Care, King’s College London, London, UK

**Keywords:** Gestational diabetes, Care experience, Pregnancy care, Emotional distress, Diabetes prevention

## Abstract

**Background:**

Gestational diabetes mellitus (GDM) is an increasingly common condition of pregnancy. It is associated with adverse fetal, infant and maternal outcomes, as well as an increased risk of GDM in future pregnancies and type 2 diabetes for both mother and offspring. Previous studies have shown that GDM can result in an emotionally distressing pregnancy, but there is little research on the patient experience of GDM care, especially of a demographically diverse UK population. The aim of this research was to explore the experiences of GDM and GDM care for a group of women attending a large diabetes pregnancy unit in southeast London, UK, in order to improve care.

**Methods:**

Framework analysis was used to support an integrated analysis of data from six focus groups with 35 women and semi-structured interviews with 15 women, held in 2015. Participants were purposively sampled and were representative of the population being studied in terms of ethnicity, age, deprivation score and body mass index (BMI).

**Results:**

We identified seven themes: the disrupted pregnancy, projected anxiety, reproductive asceticism, women as baby machines, perceived stigma, lack of shared understanding and postpartum abandonment. These themes highlight the often distressing experience of GDM. While most women were grateful for the intensive support they received during pregnancy, the costs to their personal autonomy were high. Women described feeling valued solely as a means to produce a healthy infant, and felt chastised if they failed to adhere to the behaviours required to achieve this. This sometimes had an enduring impact to the potential detriment of women’s long-term psychological and physical health.

**Conclusions:**

This study reveals the experiences of a demographically diverse group of patients with GDM, reflecting findings from previous studies globally and extending analysis to the context of improving care. Healthcare delivery may need to be reoriented to improve the pregnancy experience and help ensure women are engaged and attentive to their own health, particularly after birth, without compromising clinical pregnancy outcomes. Areas for consideration in GDM healthcare include: improved management of emotional responses to GDM; a more motivational approach; rethinking the medicalisation of care; and improved postpartum care.

**Electronic supplementary material:**

The online version of this article (10.1186/s12884-018-1657-9) contains supplementary material, which is available to authorized users.

## Background

Gestational diabetes mellitus (GDM) affects up to 5% of all pregnancies in the UK [1], between 1% and 25% of pregnancies globally, [2] and its incidence is increasing [3]. GDM is associated with an increased risk of adverse fetal, infant and maternal pregnancy outcomes including preeclampsia, primary caesarean section, excessive fetal growth (large for gestational age or macrosomia), shoulder dystocia or birth injury, neonatal hypoglycaemia, and admission to neonatal intensive care [4]. While the high blood glucose of GDM usually resolves after delivery, women with GDM have an increased risk of further episodes of GDM [5] and are seven times more likely to develop type 2 diabetes mellitus (T2DM) [6] than women with normoglycaemic pregnancies. In addition, there is growing evidence that hyperglycaemia in pregnancy has a programming effect on the long-term metabolic health of the offspring, increasing their risk of T2DM [7, 8].

The risk of adverse pregnancy outcomes can be improved by interventions directed at reducing blood glucose during pregnancy. These include self-monitoring of blood glucose, lifestyle changes and the use of glucose lowering therapies such as metformin and insulin [9–11]. However, the impact of these interventions changes the context of a women’s pregnancy to one that is highly medicalised [12, 13], as they require intensive follow-up. Current guidance from the UK National Institute for Health and Care Excellence (NICE) [14] recommends that women with GDM should be offered screening for persistent diabetes at 6-13 weeks postpartum, lifestyle advice to reduce the risk of future T2DM, and annual screening for diabetes.

In a metasynthesis of 16 qualitative studies we found that women with GDM experienced feelings of shock, upset, denial, fear, and guilt upon diagnosis, as well as a loss of normality and personal control [15]. Subsequent to this analysis several other studies have reported negative emotional effects following a GDM diagnosis [16–21], including a sense of heightened pressure and a perceived disconnect between future diabetes prevention recommendations and their own cultural practices [16, 19]. However, most qualitative studies exploring women’s experiences of GDM have not considered women’s relationships with their healthcare providers, women’s views on how to enhance care provision, and their experience of postpartum and preventive care in relation to their future diabetes risks. Of the three previous studies conducted in the UK, two included participants with mainly White ethnicity [20, 22]. However, although these studies were locally representative, this does not reflect the overall GDM population, which has a predominance of women of Asian, Black African and Caribbean ethnicity [23] and is more commonly found in socially deprived communities. In the most recent UK-based study [24] participants were more ethnically diverse, but this was a small study comprising 19 women.

In this paper we present a study that aimed to describe the experiences of women from a demographically diverse population of their GDM and GDM care, to help inform healthcare delivery for women both during and after pregnancy.

## Methods

The study used in-depth personal interviews and focus groups to elicit women’s experiences of GDM and GDM care. We obtained approval from the National Research Ethics Service (reference 13/SW/0141). We chose to use both interviews and focus groups in order to achieve a more comprehensive understanding of the data [25]. Focus groups have the potential advantage of utilising the group effect [26, 27] to explore areas of consensus and diversity in the experiences within the group [28]. Interviews have the advantage of allowing the interviewer to build trust with the interviewee as well as provide the interviewee the opportunity to analyse their own motivations, therefore improving the quality of the data [29]. In order to cater for different preferences and to widen our participation we offered participants the choice of either a focus group or an interview, and whether the interview took place on the phone, in a non-clinical building on the hospital site, or at the participant’s home.

### Setting

The study was conducted in a diabetes pregnancy unit at a large teaching hospital in London, UK. The unit currently sees around 200 women with GDM per year and has good biomedical pregnancy outcomes, with rates of macrosomia and preeclampsia lower than in the intervention arm of the ACHOIS trial [9] (a large study exploring whether treatment of GDM would reduce perinatal complications) and similar low rates of shoulder dystocia and emergency Caesarean section. To achieve these outcomes the women are intensively managed by a multidisciplinary team of diabetes specialist nurses, doctors, dietitians, obstetricians and specialist midwives with weekly or fortnightly clinic visits. The unit is based in an out-patient setting and is nested within a general diabetes clinic. This constitutes a very different context to the management of a normal pregnancy in the UK, where women are seen much less frequently and midwifery care is generally delivered in the community.

### Participants

Participants were eligible if they had had a diagnosis of GDM within the last 5 years (modified World Health Organization (WHO) criteria, [30]), were aged ≥18 years, able to speak and understand English and had a body mass index (BMI) of ≥25 kg/m^2^ (or ≥22 kg/m^2^ if Asian, in line with the Diabetes Prevention Program criteria [31]). Participants were purposively sampled based on BMI, ethnicity and area level deprivation scores, in order to ensure we included a plurality of cultural voices [32]. Potential participants were identified from clinic lists and sent invitation letters accompanied by an information sheet. To enhance participation and to ensure the sample included ‘hard-to-reach’ women we gained ethical approval to contact women directly by telephone. Participants were given a £10 voucher to acknowledge our gratitude for their time.

### Data collection

Demographic data were collected from routine medical records prior to the interviews and focus groups, and then confirmed by the participant. Participants signed a consent form, or in the case of phone interviews, gave verbal consent.

The interviews and focus groups were conducted by 2 researchers (JP, KS) in March and April 2015. We did not divide focus groups by any demographic factors, because based on our pilot focus groups, we believed that homogeneity in the group was achieved through the shared experience of having had GDM [33]. In the focus groups one researcher facilitated the discussion while the other noted down observations. The interviews and focus used the same topic guide (Additional file 1) in order to facilitate data integration. The topic guide was reflective of the study aims, addressing women’s experiences of GDM and the care they received, and was piloted prior to the main data collection. The researchers actively probed responses to elicit in-depth accounts of the women’s views and experiences, following Rubin’s methodology [34]. The focus groups were similarly structured and the researchers managed the groups to ensure all women participated and voiced their experiences. The interviews took place on the telephone (*n* = 7), in participants’ homes (*n* = 5), in a non-clinical room at the hospital (*n* = 2) or at the participant’s workplace (*n* = 1) and lasted between 20 and 120 min. The focus groups were held in a non-clinical meeting room and lasted between 60 and 120 min.

### Data analysis

The interviews and focus groups were digitally recorded, transcribed verbatim and imported into NVivo version 10 for analysis. Framework analysis [35] was used to analyse the data. This approach allows themes to develop both from the research questions and the transcribed texts. It also ensures a close link to the data is maintained throughout the analysis process, it allows data to be explored both by theme and in the context of each individual (or focus group), and its highly structured and systematic process allows for data to be analysed by more than one researcher [36]. The approach was also chosen for its flexibility, as it is not strongly linked to a particular theoretical approach [36], other than subtle realism [37]. The data were analysed in 5 stages following this approach:

1. Familiarisation – at this stage two researchers (JP, KS) read all the transcripts repeatedly in order to familiarise themselves with the raw data.
2. Identifying a thematic framework – three researchers (JP, KS, AF) independently coded a selection of transcripts, and then met to discuss the codes. From this they developed an initial thematic framework.
3. Coding/indexing – the framework was then applied systematically and continually revised until the data from all the transcripts were captured. A codebook of themes was developed and modified by the researchers following an iterative process. Two researchers (JP, KS) coded all the transcripts and met frequently throughout the process to address any discrepancies. A third researcher (AF) then moderated the codes and addressed any uncertainties or ambiguities.
4. Charting – after coding, the data was entered into framework matrices for each theme in NVivo.
5. Mapping and interpretation – during the interpretation phase we detected elements of the data in order to group data into categories and identify key dimensions, as described by Ritchie et al. [38] These key dimensions became the main themes of the results.

The final component of the analysis was to translate the themes into potential theoretical models to describe the experiences of women during a GDM pregnancy.

## Results

From a total population of 614 women with a GDM pregnancy between 2010 and 2014, 536 met our inclusion criteria based on their medical records. We were able to contact 118 of these women, and 78 (66%,) agreed to participate. Reasons for non-participation were: unavailable on the dates offered (*n* = 9), not interested (*n* = 8), requested more information but were later uncontactable (*n* = 7), did not meet inclusion criteria (n = 7), overseas (*n* = 2), too busy (n = 2), still angry about care received (*n* = 1), subsequently diagnosed with type 1 diabetes (n = 1) and long term illness (n = 1). Twenty eight of the women did not attend their interview or focus group session. A total of 50 women took part (15 were interviewed and 35 attended one of six focus groups). The participants were representative of the eligible population of women with GDM attending the clinic in terms of age, ethnicity, BMI, and parity (Table 1).

**Table 1:** Demographic characteristics of participants compared to the clinic population

	Participants	Clinic population
Age		
Mean (years)	37.7 (range: 21-53)	36.5 (range: 19-53)
Ethnic group		
Black	50%	54%
White	26%	27%
Asian	18%	14%
Mixed	6%	2%
Other	0%	2%
BMI		
Mean (kg/m^2^)	34.7 (range: 25-49)	33.0 (range: 21-57)
Deprivation rank		
Mean (IMD)	7688	8462
Parity		
Mean (number)	2.2	1.4
Primiparous (%)	31%	17%
Multiparous (%)	69%	83%

### Themes

Seven main themes were identified: the disrupted pregnancy, projected anxiety, reproductive asceticism, women as baby machines, perceived stigma, lack of shared understanding, and postpartum abandonment. These themes are presented below together with excerpts of supporting data.

### The disrupted pregnancy

Many women in this study found the diagnosis entirely unexpected, and the immediacy of enforced dietary change, pharmacological treatment, frequent hospital visits and self-glucose monitoring overwhelming and frightening: ‘*I didn’t understand very clearly why it happened all really, really quickly. It was quite difficult to get my head around it really’* (Interview participant (IP) age 27, 4 children, mixed ethnicity).

This disruption left women *‘in complete shock’* and extremely scared of the possible effects on the baby. They felt panicky, *‘freaked out’* and some burst into tears. Two women described leaving the hospital immediately upon diagnosis without waiting to see the dietician: one explained, *‘I think I was so overwhelmed I just wanted to get out of there’.* Another described the intense experience of diagnosis:

*‘It was like really intense, you know, I’d gone to [the hospital] just for a booking in appointment with the midwife and then immediately I was sent through to a dietician and then the dietician said, ‘from now on you’re going to be just with this team and this team will make sure that this happens and that happens and the baby’s at risk of this and that and people who have gestational diabetes, some have babies die’* (IP age 44, 2 children, Black British).

Women identified that an earlier warning about their GDM risk would have ameliorated the shock of GDM and also allow them the opportunity to make dietary changes sooner.

Conversely, a few women did not find the diagnosis shocking or particularly emotive. Some women did not believe the diagnosis (*‘I believe I don't have [diabetes], I don't know, but I'm not diabetic’* (IP age 45, 6 children, Somali)), and another *‘I was shocked and not shocked… The reason I wasn’t shocked is because I was warned that, you know, your parents have got diabetes, it might be hereditary’* (IP age 52, 3 children, other Asian).

### Projected anxiety

Women’s experiences of GDM care and their interaction with healthcare providers were often a complex combination of feeling well cared for yet over-scrutinised. This perhaps reflects the level of responsibility and anxiety felt by healthcare providers in achieving a good clinical outcome for the baby in a limited time frame. Participants discussed being closely monitored and given difficult and sometimes threatening information about their baby’s risk. While many women found this level of attention reassuring and liked the fact it meant that they *‘had to behave’*, they also found it difficult:

*‘I know they are helping me but, I mean, they are very strict. Honestly, any time I got pregnant I went to do the GTT* [oral glucose tolerance test], *I said to myself ‘oh please God let it be negative, I don’t want to go and see these people’, because you can’t miss them or they will chase you. They will chase you… [This] scare [sic] a lot of people...we all want to be free, do our own thing, it’s not like some people telling us what to do’* (Interview participant (IP) age 42, 4 children, Ghanaian).

Women also described how the healthcare provider’s anxiety sometimes manifested itself in a negative discourse where they felt chastised by the health professional if they were non-compliant: *‘So we’re already stressed. And now you’re going to shout at us for not bringing the monitor and having a KFC...it just feels like you’re getting in trouble’ *(Focus group participant (FGP) age 30, 2 children, Black Caribbean). This interaction compounded the women’s anxiety and sometimes led to disordered eating, as the same participant described: *‘I’m a comfort eater so when I’m upset I have to eat...There was one particular day I remember and she [the healthcare provider] really upset me and I went straight to the canteen’.*

Overall, the demands exerted on women by the healthcare providers to comply with the diet and treatment regime were stressful for many: *‘The whole pressure with the whole everything, it really did affect me and I think it’s probably one of the worst times I’ve had in my life actually’* (IP age 39, parity unknown, Black British). For some women, this experience was so traumatic that it prevented them returning for a postpartum glucose test: *‘I know I need to keep getting checked, but I don’t, because of how bad my experience was of it: I don’t want anything to do with it’ (*FGP age 28, 1 child, Black British*).* A few women did not find the experience stressful at all, as one participant reported: *‘I felt very normal throughout the pregnancy; there was no bad experience during the pregnancy’* (IP age 33, 3 children, Nigerian).

Women were also subjected to the healthcare providers’ anxiety during and immediately after the birth, as one participant described: ‘*…my baby’s feet were like black and blue from all the poking, and the [midwife] cried… She couldn’t do it. She had to compose herself and then come back and do it. I mean I was crying anyway but it was such a horrible experience’* (IP age 27, 4 children, mixed ethnicity). Overall the impression given by the women was that there was intense concern expressed by the health care professionals for the baby’s welfare, which led to heighted sense of pressure on the women and made many very anxious and fearful.

### Reproductive asceticism

Women felt emotionally challenged by the need to observe a strict code of personal behaviour and externally imposed asceticism to ensure a healthy infant. This often led to negative reflections on their health behaviours prior to their diagnosis: women mentioned *‘beating themselves up’* and wondering if the condition was due to the fact they had ‘*never been able to resist chocolate’*. One said, ‘*I felt kind of, I guess, disappointed in myself that I’d let things get to that stage’* (IP age 39, 2 children, Sri Lankan), and another*: ‘All I could think about was how I’d damaged my baby’* (IP age 34, 1 child, Indian)*.*

Some of the behavioural requirements placed on the women, particularly self-monitoring of blood glucose, resulted in feelings of failure. Several women felt distress at having to monitor their blood glucose, *‘a test you're potentially failing four times a day’ (*FGP age 43, 1 child, Greek*),* and described it as the worst aspect of having GDM. Participants struggled with the fact that their blood glucose readings were often high, in spite of their abstemious efforts.

*I wouldn't want to look, I’d make my husband look, like ‘what does it say?’, and I got so sad I just stopped eating... I was crying all the time and I just stayed in bed for ages and there was like a big fear...that was the saddest part, having to check, yeah, four times a day, it was just hard* (FGP age 28, 1 child, Black British).

Conversely, some women did not alter their behaviours following GDM diagnosis and continued as before, as one participant described: *‘I’ll be honest. I didn’t take no notice. I ate what I wanted, I done what I wanted and my baby’s fine’* (FGP age 30, 2 children, Black Caribbean).

The burden of responsibility of maintaining the intense regimen to lower their blood glucose provoked an emotional response both in relation to women’s beliefs about how they have caused their GDM and to their own self-censure as they struggled to accommodate the new behaviours demanded of them or when they failed to achieve the suggested diet and glucose targets. Much of these emotions were reflective of the feared consequences for their baby.

### Woman as baby machines

Participants described feeling unimportant in the GDM process, and some were aggrieved by the lack of autonomy they were permitted in relation to their pregnancy: *‘anything you might think, it’s not really important’* (IP age 39, parity unknown, Black British). One participant described how the threat of harm to the baby was held over her to secure compliance:

*‘I think because I was resisting it and I was told by a midwife that basically, you know, ‘if you try and resist they will throw everything at you if you try and take back a bit of autonomy and a bit of control, they will throw the book at you’. I was constantly being told ‘if you don’t do what we say your baby might die, the baby might die, the baby might die…’ I felt it was about control, because also the evidence was what I was doing was working’* (FGP age 43, 1 child, Greek).

Indeed, the message received by the majority of participants from the healthcare provider was that the focus of concern was for the baby – *‘it was kind of more drilled into me that it was for the sake of your baby more than for you’* (IP age 27, 4 children, mixed ethnicity) – and the woman was a possible obstacle to the baby’s wellbeing. One participant described the care team as having the attitude that: *‘you might ruin that baby, we don't want you to ruin that baby’ (*FGP age 28, 1 child, Black British*).* Women discussed feeling that the hospital claimed ownership of the baby: *‘my husband sometimes says, “Have you noticed the [hospital’s] behaviours, it’s not your child it’s their child!”’* (FGP age 36, 2 children, Bangladeshi). Another participant described her experience of a lack of bodily ownership during her pregnancy: *‘In the end you just feel like you’re a dead person walking with a baby inside you, do you know what I mean? Like all these terrible things are going to happen to you’* (IP age 42, 2 children, Black British).

Overall, women felt they were viewed objectively rather than personally, with the balance of control residing with the healthcare providers.

### Perceived stigma

Women perceived that they were stigmatised for having GDM. In addition to the self-blame they experienced as a result of enforced reproductive asceticism, they felt external recrimination for their condition and believed they were not trusted by the healthcare provider. This stigma was perceived from both society in general (‘*all I would see on TV is if you’re overweight, you’re obese, you get diabetes and it’s your own fault kind of thing. Even when I had gestational diabetes I felt like oh gosh, I’ve got gestational diabetes, you know, people are looking at me like oh, I’m overweight, that’s why I’ve got it’* (IP age 27, 4 children, mixed ethnicity)) and from healthcare providers *(‘I often got the feeling that health professionals thought that I just hadn't been thinking about my health or taking care of myself or putting any effort and thought into it’* (IP age 39, 2 children, Sri Lankan)). Consequently, women reported that their blood glucose readings were often not believed by health care providers: ‘*Sometimes, you know, if you're in a good mood you just laugh, you say, ‘you don’t believe me?!’* (IP age 42, 4 children, Ghanaian).

The stigma felt by the women was exacerbated by being labelled as ‘diabetic’ as opposed to ‘pregnant’, and by being treated in a diabetes setting rather than a usual maternity setting. Some requested to be treated separately from people with long-term diabetes:

‘*I think that would have made me feel like I was being treated more like a pregnant woman in the round rather than someone with a lifelong condition who hasn’t been controlling it*... *Yeah, ‘you're a diabetic, go with the diabetics over there, go to the diabetic clinic and you'll be seen with all the other diabetics and that’s what you are now, you're a diabetic’* (IP age 39, 2 children, Sri Lankan).

Many participants discussed ways in which they felt judged. For example, one woman described how assumptions were made based on her cultural background:

*‘sometimes they look at your cultural background and, two people told me things like, ‘Oh, stay away from fried chicken and rice and peas.’ And it’s like, hang on a second. For a start, I don’t eat fried chicken… it did just feel a bit, kind of, like, wagging finger syndrome. It was a case of, ‘No, you don’t know me, you can’t look at my postcode, my age and my ethnic background and know about me and give me advice on that.’ So I found it maybe a little bit patronising’* (FGP age 43, 3 children, Black British).

The experience of stigma is identified as being labelled as a person who, through their own failings, has brought the condition upon themselves. It is experienced as a reduced sense of autonomy with high levels of external control and judgmental interactions.

### Lack of shared understanding

Women sometimes reported a lack of shared understanding between patient and healthcare provider. This was both in terms of participants’ lack of comprehension of GDM, and their perception that the healthcare provider did not fully empathise with their position. The shock of the GDM diagnosis coupled with the complex nature of its management left many women unable to digest information routinely given to them by healthcare providers, which resulted in confusion and further feelings of not being in control. One participant explained, *‘probably things were there [that] I don’t understand were there at the time. I didn’t understand what they were talking about’* (IP age 44, 4 children, Black Caribbean).

Participants often perceived that the healthcare providers did not understand them, which again, lead to feelings of alienation, loss of trust and deceitful actions. One participant described her relationship with the dietitian:

*‘She didn’t understand. She couldn’t possibly have understood because I said, ‘have you got kids?’ and she says ‘no’. OK and you clearly don’t have an eating habit, or you just have a metabolism that is faster than mine. I just felt a bit - she used to say ‘what did you have for lunch?’ and I’d think ‘carrots, broccoli, a small portion of rice’ and I’d had a KFC! I can’t tell her that because I’d feel a bit embarrassed...I just think it’s a bit err, going to see a very slim, perfect body lady and then there’s me’* (FGP age 30, 2 children, Black Caribbean).

This incongruity between the women and their healthcare providers often manifested as a perceived lack of attention to the participants’ individual needs. Much of the discussion around this focussed on women’s views that the dietary advice they received was culturally irrelevant to them. One participant explained:

*‘*The issue that I had was as a vegetarian. The person who was monitoring me, she didn’t have much knowledge of Asian vegetarians’ (IP age 52, 3 children, other Asian).

A few participants mentioned not being able to eat the small portions advised, as it was out of keeping with their culture:

*‘We black, we Jamaican, we go the wrong way eating things!...When I look on the sheet and it’s like one scoop of rice, but it, kind of, the food balance is, to me, it’s kind of too small… I have a problem with the diet … I can’t eat veg every day like a lot of people, especially White, when they eat, their plate all the veg! And I find that wooooh!’* (IP age 44, 4 children, Back Caribbean).

This lack of perceived understanding led to a loss of trust, and patients’ confusion about GDM potentially exacerbated their feelings that they were not in control of their pregnancy choices.

### Postpartum abandonment

In pregnancy, most women reported feeling very well cared for, in spite of their negative experiences: *‘the diabetic clinic, they clearly were very supportive’* (IP age 48, 1 child, other White). However, many women reported very little postpartum follow-up. Women found this surprising, considering they had been told they were at risk of diabetes: ‘*I had a really intense relationship... and then it was just cut, and it was like ‘now you’re fine’, but I’m not fine coz I’m told I’m at risk’* (IP age 38, 2 children, White British). Another woman explained, *‘Once you’ve had your baby it’s on your way mate, we’re done with you. They don’t bother... Once they’re done with your baby they’re done with their job’* (FGP age 38, 4 children, British Indian).

Women generally wanted to be followed up and not to be *‘forgotten’*. Several participants had subsequently been diagnosed with impaired glucose tolerance or T2DM and, in contrast to the pregnancy, had received no support. One participant with subsequent T2DM described how the healthcare provider had the power to help her but did not, now she was no longer pregnant:

*‘instead of you [the healthcare provider] waiting ‘til me come and beg you to say, ‘I can’t lose the weight and I’m desperate’, and then all they put the blame on you like you aren't doing anything while they know everything… them have the money, them know how to do it and whatever, like we don’t know how to do it, we have to ask’* (IP age 44, 4 children, Back Caribbean).

Women described the lack of support for breastfeeding, resulting in guilt and feelings of disconnection from their babies. They described being encouraged to bottle or mix feed due to infant hypoglycaemia, and believed that staff did not prepare them for this nor spend time supporting them with breastfeeding *‘because it’s convenient for themselves’*:

***FGP age 28, 1 child, Black British*** *: I’m sure they see women who have gestational diabetes give birth, and then the issue with baby having low blood sugar, it must happen quite frequently. I mean, we've all had it, so you would think they would then look at that breastfeeding, because that is a massive thing for women just given birth who want to be able to feed your baby. If we took it to heart in terms of ‘I want to breastfeed’, that absolutely killed my morale, absolutely completely. It took months to get over that.*

***FGP age 21, 1 child, Black British*** *: I stood over the kettle and cried. Making bottles and it was just like this isn't what I planned, you know.*

***FGP age 28, 1 child, Black British*** *: Yeah, completely. And now it’s OK but there was no link almost, there was no information to try and make you feel like [breast feeding] was possible.*

This postpartum abandonment seemed to compound the message perceived by women that they were not important: their job was to do as they were instructed to produce a healthy infant and once this task was achieved they were redundant and no longer of interest.

## Discussion

Clinicians and women with GDM are under immense time pressure to control blood glucose levels in order to produce a healthy infant. This leads to a somewhat paternalistic healthcare context, where information and decisions are largely made by the healthcare provider on the assumption that health – in this case that of the baby – is the priority rather than personal choice and control. This is in contrast to current thinking about models of patient-provider interaction, where the paternalistic model is largely considered to be justified only in emergency situations [39], and principles of shared-decision making [40] and motivational enhancement [41] are generally favoured. The women’s accounts relay a sense of alienation in which they are reduced to biological vehicles for the production of infants. The impact of medicalisation of women’s care in high risk pregnancies on their autonomy has been reported previously. Furber et al. [42] found that obese women felt ignored as a result of excessive screening, and described feeling like ‘an oven’. In addition, Figuera et al. [43] found that women with GDM felt out of control as a result of the lack of choice and ‘cascade of interventions’. While this model has resulted in very good clinical outcomes with low levels of complications, it comes at a cost to the women, heightening their emotional distress. Some women seem to resist this care approach and in the longer term, it may enforce a sense of ambivalence toward their own health.

A key theme is the sense of abandonment felt after delivery, as the period of intense intervention ends and women are left uncertain about addressing their diabetes risk. A similar theme was identified in Morrison’s study on Australian women’s experiences of GDM [17] et al., where women described ‘no one caring’ after their pregnancy was over. Aside from the missed opportunity to follow up women to address their future diabetes risk, research from other fields shows that sudden abandonment after a period of intensive healthcare can result in feelings of vulnerability and a loss of importance [44]. This would suggest that more attention needs to be given to the emotional distress that accompanies a GDM pregnancy and to postpartum support. However, it is important to note such a change should not compromise the clinical outcomes, especially as the women themselves value the efforts of the clinical team to help ensure their baby is healthy.

Another factor that appears important in driving the distress is women’s sense of personal responsibility for the GDM. They feel guilt and shame that their previous behaviours may have contributed to their GDM, and once diagnosed, experience further guilt for failing to control blood glucose levels. This observation is strongly concordant with previous studies of women’s experiences of GDM [15, 17, 19, 45, 46]. Similarly to previous studies [15, 19, 47], our participants were often shocked by the GDM and did not understand why they were not warned of the risks sooner, such that they might have modified their behaviours. While this would seem a logical request it is important to note that interventions in early pregnancy have to date not shown significant reductions in GDM [48]. Nevertheless, women may benefit from being prepared for the potential of GDM to both diminish the shock and to start to adapt their health behaviours in advance.

It is also noteworthy that many of the women either struggled with or resisted the self-management behaviours recommended in GDM. This resistance and struggle has been noted in previous studies. Carolan et al. [47] reported that women with GDM wanted more time to adapt to the new dietary restrictions and in Ghaffari et al.’s study [19] women struggled with dietary changes and administering insulin, due to lack of autonomy. The women’s accounts suggest there are a number of factors – both emotional and structural – that explain why the behaviour changes are so burdensome. From an emotional perspective, the requirement to make immediate behaviour changes in response to a surprise diagnosis of a largely asymptomatic condition means women do not have time to adapt. Kubler-Ross’s model of grief [49] indicates that upon diagnosis of a disease, people often transition through several stages before accepting their diagnosis: denial, anger, bargaining and depression. This takes time that, in the case of GDM, women are not afforded. Some women with GDM have not accepted their diagnosis and are in the denial phase of Kubler-Ross’ model. Such an outlook may be characterised by avoidance behaviours and enforce a passive status on the women, reinforcing the controlling behaviours of the healthcare providers.

Other women respond to GDM with fear. They accept their diagnosis, assuming their baby is at great risk *(“I took it to be sort of, like, life and death”,* (IP age 39, parity unknown, Black British)) and follow instructions to their best abilities. In Evans et al.’s [50] study women with GDM experienced a sense of confusion and failure when blood glucose readings did not reflect the perceived effort they were putting into their diet. Many of these women ‘starve’ themselves, a phenomenon also reported by Draffin et al. in their recent study [24] and are deeply traumatised by self-blame, failure to achieve blood glucose targets and fear of what might happen to their baby. This leads to feelings of depression and isolation, and sometimes a complete disengagement after the birth. These women display unresolved feelings of trauma and some did not attend the postpartum glucose tolerance test, in spite of the belief that they were at risk of diabetes, because the trauma renders them wholly averse to any further connection with dieting, diabetes or diabetes care. According to Kubler-Ross’ model, these women could be in the bargaining or depression stages of acceptance.

From a structural perspective it would seem that some of the challenges experienced by the women were products of their relationship with the healthcare provider and the way this was constructed in the process of care. While many women feel supported and cared for, others seem more resistant to the provider’s efforts to help them. They see no benefit to the close monitoring and strict approach they are subjected to, and are not motivated to comply with their treatment except to avoid being chastised. Some actively reject care by not taking medication and lying about food intake and blood glucose readings. Some participants wrestle for control with the diabetes clinic, and are sceptical about information they receive. The underlying cause of this response is unclear, although it is notable that unlike women with type 1 diabetes [51], these women present the demands as coming from the healthcare team rather than from their baby. A few women reject their diagnosis or their care entirely. They feel aggrieved at the lack of autonomy they are given over decisions about medication, and believe their views and feelings are not taken seriously. These women find the monitoring and lack of trust from the healthcare provider extremely undermining and lose faith in their clinical care. This rejection often results in anger, and therefore a complete disengagement after the birth. This range of reactions to care is reflected in Morrision et al.’s survey of 393 women with GDM [17], which uncovered great variance in perceptions of seriousness of GDM, levels of adaptation and reactions to the care provided.

While we have not included data from health professionals in this paper, it may be that the context of care experienced by the women is a product of the relationship between the health professionals and the women. Due to the potential health implications for the baby and the time pressure, healthcare providers are unsurprisingly anxious to avoid complications, and this anxiety may be projected onto the patients through the use of threatening or frightening language about the risks to their baby alongside intensive behavioural monitoring. This projected anxiety, coupled with perceived stigma around GDM, and the alienation women experience from a normal pregnancy results in a traumatic experience. This includes feelings of isolation, depression, guilt, fear and confusion, and can lead to resistance expressed as non-compliance, deceit and disordered eating.

Pregnancies in general are becoming more medicalised as society is becoming more risk-focused [52], which can overemphasise the adoption of controlling behaviors from health providers at the cost of the women’s personal autonomy. Feminist theorists have described the phenomenon of the ‘public fetus’, where recent technologies that allow us to view images of the fetus have led to them being seen as a separate entity from the mother, rather than part of her [53]. This combination results in a model of antenatal care where medical experts and societal pressure encourage reproductive asceticism [54], and Lupton [52] described how in this scenario, the pregnant body has also become public property, subjected to judgements and criticism. This would seem to resonate with women’s experiences of GDM care, as the women felt so removed from the focus of their care that they described the hospital as the owner of the baby, as if they had handed over all rights to their body. The seeming unimportance of the woman as an individual in her own right is re-emphasised following the birth of her baby, when the intensive level of care abruptly ceases. Further to this, those women who were later diagnosed with T2DM or impaired glucose tolerance described receiving no support, in comparison to the high level of support provided during pregnancy. This again reinforces the message that the woman is of little importance, and her body is of no interest past carrying the baby.

Societal expectations of reproductive asceticism, as described, can lead to judgements and criticism. This is exacerbated in GDM, where women are stigmatised for their ‘choice’ to be overweight [21]. Research shows that there is general stigma against people with T2DM and those who are obese [42, 55]. When the ‘public fetus’ is seen to be affected by the woman’s individual choice, the stigma is intensified, which can lead to feelings of guilt, depression and negative self-beliefs. This, coupled with the trauma of the GDM experience and little acknowledgment of the woman’s individual worth can only intensify the difficulties women face addressing lifestyle choices for themselves after birth. Therefore, in spite of being able to achieve excellent clinical outcomes for the baby in the short term, the current UK model of GDM care (coupled with societal influences) may be at the cost of poor psychological and longer-term clinical outcomes for the woman, who may not re-engage with addressing her future diabetes risk.

Many of the findings from this study are concordant with those from previous studies on women’s experiences of GDM from a number of different countries. However, this study adds to current research by providing an extended analysis of women’s experiences in the context of the model of care, drawing a possible link between the experiences of GDM and long term psychological and potentially clinical outcomes.

### Translating women’s experiences of GDM

**Fig. 1:**
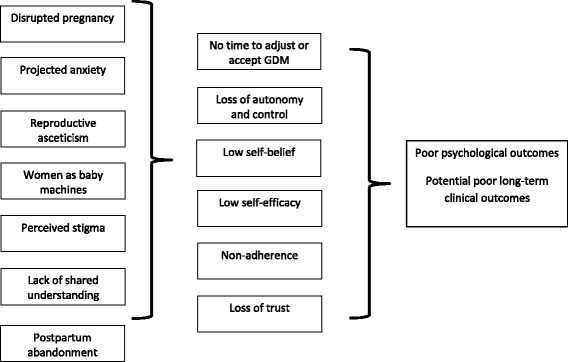
Relationship between women’s experiences and potential care outcomes

In the final part of our analysis we integrated the themes into a collective model to hypothesise a potential relationship between women’s care experiences and personal health outcomes. This model is presented schematically in Fig. 1.

The model details how the themes we have identified may translate into health outcomes. The women’s experiences can be damaging and the context of their experiences mean that women have little time to adjust to what is happening. They also have reduced personal autonomy and can lose self-belief and experience low self-efficacy as they struggle to fulfil all the demands placed on them. This struggle can damage their relationship with and trust for their healthcare providers and lead to disengagement and avoidance behaviours. Collectively these experiences seem to have an enduring effect on the women’s views of their own health. This endurance may be related to the legacy of the emotional distress experienced and the lack of time available for women to go through the stages of grief relating to their ‘transient’ disease state, all of which may be problematic in the context of managing their own future risks of diabetes.

In light of these observations, healthcare delivery may need to be reoriented to enhance the pregnancy experience and to help ensure women are more engaged in their care and attentive to their own health. Areas for consideration may include: identifying women at high risk of GDM in early pregnancy and providing information, so that the diagnosis is less shocking; improved management of the emotional consequences of a GDM diagnosis; a less judgemental and more motivational approach to enhance the women’s self-efficacy and engagement in their care; consideration of the context of care to deliver a more ‘normal’ and positive experience of pregnancy, such that the women feel important and their needs are more central; and improved postpartum follow-up so that women do not feel neglected after the birth and are supported in attending to their own ongoing health needs in the context of adjusting to motherhood. However, it should be cautioned that any changes made would need to be balanced against the fact that the current model results in good clinical pregnancy outcomes.

### Study strengths and limitations

A major strength of our study is that while study participants were, as in all similar studies, self-selecting, the participants demographically matched the overall clinic population. This population is demographically diverse and participants represented a wide range of views and feelings. Another strength of our study compared to many similar studies is that we offered participants the option of an interview or a focus group, whereas many studies use only one methodology, thereby biasing participation based on women’s preferences. It should be noted that there are some potential differences between the results of telephone and face to face interviews, for example telephone interviews are not able to benefit from visual cues so it may impact rapport [56], although this does not always impact findings [57]. It is possible that holding interviews on the hospital site also influenced participants’ responses due to its proximity to the place the participant received their care.

It should be noted that the generalisability of our study may be limited, as our participants were identified from a group of women attending one hospital in southeast London, UK. Therefore the findings may not be applicable to other population groups using different models of care. In addition, our sample was self-selecting, and therefore may not represent the views of the population studied. However, as stated above, our sample was representative of our population group in terms of ethnicity, postcode deprivation index, age and BMI. In addition, many of the themes elicited articulate social, cultural and psychological situations that are likely to be applicable in contexts outside of the UK, and indeed reflect findings from previous studies conducted in a variety of countries. This indicates that the issues discussed may be widespread.

Another limitation implicit to qualitative studies is the possibility of researcher bias in interpretation. To mitigate this, three researchers were deeply involved in the data analysis, two of whom independently coded every transcript and then agreed codes and themes, with the third researcher moderating the process.

## Conclusions

GDM is an emotionally distressing experience for many, although not all, women. While most women were grateful for the intensive support they received during pregnancy, the costs to their personal autonomy were high. Women described feeling valued solely as a means to produce a healthy infant, and felt chastised if they failed to adhere to the behaviours required to achieve this. This sometimes had an enduring impact to the potential detriment of women’s long-term psychological and physical health. Healthcare delivery may need to be reoriented to improve the pregnancy experience and help ensure women are engaged and attentive to their own health, particularly after birth, without compromising clinical pregnancy outcomes. Areas for consideration in GDM healthcare include: improved management of emotional responses to GDM; a more motivational approach; rethinking the medicalisation of care; and improved postpartum care.

## Additional file

Additional file 1: This file is the topic guide used by the researchers for the interviews and focus groups. (DOCX 25 kb)

## Abbreviations

(BMI): Body mass index
(FGP): Focus group participant
(GDM): Gestational diabetes mellitus
(GTT): Glucose tolerance test
(IP): Interview participant
(NICE): National Institute for Health and Care Excellence
(T2DM): Type 2 diabetes mellitus
(WHO): World Health Organization
