# Supplementary material for: Experiences of gestational diabetes and gestational diabetes care: a focus group and interview study
Source: BMC Pregnancy Childbirth. 2018 Jan 11;18:25. doi: 10.1186/s12884-018-1657-9 (PMC5765597; doi:10.1186/s12884-018-1657-9)
Supplement: Additional file 1: — This file is the topic guide used by the researchers for the interviews and focus groups. (DOCX 25 kb) [file 12884_2018_1657_MOESM1_ESM.docx]

**Interview and focus group topic guide**

**Overview, aims and consent**

[Researcher does introductions and explains the research, the aims of the interview/focus group and what will happen. Researcher then goes through the consent form with participant(s) and asks them to sign it (or give verbal consent if interview is over the phone), having checked participants’ understanding]

**Experiences of gestational diabetes**

**Aim: to find out participants’ experiences of gestational diabetes**

1. We would like to find out about your experiences of having diabetes in pregnancy. Perhaps you can tell us a little bit about this.

Prompt questions:

* How did you feel when you were told you had gestational diabetes?

* Can you tell me a bit more about how that affected you?

* [if participants says it was very stressful etc.] That must’ve been really hard for you. Was there anything that helped you with all that worry?

* Anything else about your experiences?

**Pregnancy support**

**Aim: to identify what kind of support participants would find helpful during pregnancy**

1. When you were pregnant did anyone explain to you about the potential for developing diabetes later on?

Prompt questions:

* [if no] Would you have found this information helpful?

* [If yes] Can you tell me a bit more about any information you’ve been given?

* Were you given any information about activity and diet when you were pregnant?

* What information were you given about activity and diet?

* Was any of this information helpful?

* Were you able to follow the advice you were given?

* What was it that made it helpful / unhelpful?

* Anything else?

**Post-pregnancy support**

**Aim: to identify what kind of support participants would find helpful in the initial postpartum period**

1. Having a healthy diet and being active can reduce your risk of getting diabetes in the future. Obviously, this is not easy, especially with a baby or with young children, and we are trying to find ways we can support women who would like to do this. Thinking back to the first couple of months after you had your baby, were you given any advice or support with weight loss during this period?

Prompt questions:

* What do you think would be helpful in that first few months?

* [After participant makes a suggestion] How do you think that would work with the baby / what are the practicalities of that?

* [After participant says 'I'm too tired' or 'I don't have time'] Yes, it is really challenging.  It would be helpful for me to know a little bit more about that - what is it exactly that stops you?

* [If participant says 'I am / was not interested'] Ok, it sounds like this is something you haven't thought about or isn't of interest for you.  What do you think would be helpful for women in your position who were interested?

* How frequently should what you suggest be provided?

* Who do you think should provide this support?

* [If participant says ‘I’m just too tired’, or ‘I’m too lazy’] Yes, it is obviously very hard. What would have to happen for you to overcome the tiredness?

* Anything else?

**Post-infancy support**

**Aim: to identify what kind of support participants would find helpful in the post infancy period**

1. So we have talked about the first few months after having a baby.  Moving on, were you given any advice or support with weight loss after this point?

Prompt questions:

* What do you think would be helpful in that first few months?

* [After participant makes a suggestion] How do you think that would work with the baby / what are the practicalities of that?

* [After participant says 'I'm too tired' or 'I don't have time'] Yes, it is really challenging.  It would be helpful for me to know a little bit more about that - what is it exactly that stops you?

* [If participant says 'I am / was not interested'] Ok, it sounds like this is something you haven't thought about or isn't of interest for you.  What do you think would be helpful for women in your position who were interested?

* How frequently should what you suggest be provided?

* Who do you think should provide this support?

* [If participant says ‘I’m just too tired’, or ‘I’m too lazy’] Yes, it is obviously very hard. What would have to happen for you to overcome the tiredness?

* Anything else?

**Prioritising support**

**Aim: to identify priority elements of support**

1. Thanks, I think we have identified some really important ways that women can be supported to stay healthy after diabetes in pregnancy, including x, x and x.  We have also thought of a few other things that might be helpful.  These are:

* Lifestyle coaching (working with someone one to one to look at your lifestyle, face to face or on the phone or a mixture of the two)

* Groups (could be anything from weight loss groups, cooking classes, exercise groups, information groups, support with diabetes groups)

* Web forum (an online forum where women who have had gestational diabetes can chat to each other, or get an advice from an expert)

* Phone app (these can include all sorts of things from food and exercise diaries, to brief exercise programmes, to menus etc.)

* Meal planners and cards (these are paper-based cards that help you plan meals

* Leaflet (with information about diabetes, how to stay healthy, or where you can get support)

* Text messages (these could provide tips about eating for example, or encouragement, or links to information online, or quiz questions for example)

* Food and exercise diary (where you record what you have eaten and the activity you have done)

* Pedometer (which you wear and it measures how many steps you walk a day)

1. So, thinking about all of the things we came up with, plus these others, what do you think are the most helpful things to support you?

Prompt questions:

* Why do you think it is helpful / not helpful?

* Do you think that X would be beneficial for anyone else / everyone? Or are there some people who would not benefit?

* Where would be a good place to provide X? For example, at the hospital, GP practice, a community centre or children’s centre, somewhere else.

* What do you think would be the most helpful things we have discussed?

* Which would be the least helpful?

* Is there anything else you would like to say or ask about?

**Summary**

**Aim: to explain next steps and give participants their vouchers**

[Researcher explains what will happen next, where to access further support and gives participant their gift voucher]
